# Editorial

**Published:** 1864-02

**Authors:** 


### Editorial.

### A CHLOROFORM CASE.

Mrs. B. called, Feb. 16th, to have some fifteen teeth extracted. The operation was to be performed at 2 P. M., she having eaten nothing since early breakfast. The teeth were very firmly set, and all, as found on extraction, affected with exostosis. She inhaled pure chloroform, without anything unusual occurring till all the upper teeth and two of the lower were removed. As I turned to change forceps, she ceased to breathe. Supposing the *function* of respiration to be overcome by the anæsthetic, I immediately resorted to artificial respiration, but found that, though I could force air out of the lungs, none went into them. I immediately concluded that the difficulty arose from mechanical, or spasmodic closure of the glottis. The patient's face had, by this time, become as dark colored as that of a person in epilepsy. Something had to be done. I forced the index finger down the throat, as far as practicable, made firm, sudden pressure on the base of the tongue, tipping the head suddenly forward at the same time, and had the satisfaction of finding the patient instantly relieved. A large, firm clot of blood, appearing to have been molded between the tongue and the roof of the mouth, came forward, over the finger, and explained the whole difficulty. After a few full inspirations, the operation was completed by the rapid removal of six more teeth, and the patient was laid in a horizontal posture, and, in a few minutes, was fully conscious, and had no unpleasant symptom afterward. To-day I saw her; and she is delighted with chloroform.

Is it probable that some of the fatal chloroform cases have resulted wholly from strangulation? This case would have proved fatal, beyond a doubt, if the nature of the difficulty had not been almost immediately recognized and acted on. I would rather give ether or chloroform for any other class of operations, than for the extraction of teeth.

W.

### OHIO DENTAL COLLEGE ASSOCIATION.

Our Alma Mater, the Ohio Dental College, is owned, as most readers of the Register know, by the above named Association. At a former annual meeting, committees were appointed to secure stock sufficient to liquidate the debt on the college property. These committees, at the late meeting, reported complete success. The stock is raised, and by the next annual meeting will, no doubt, all be paid in. The profession may well feel proud of such an achievement. The college building now stands as a monument of the enterprise and disinterestedness of the profession. By a unanimous vote, the stock was made "non-interest bearing." This will be a relief to the overburdened faculty, all of whom held their positions at a heavy sacrifice, if the value of time is taken into consideration. But in their devotion to the cause of science, we have another illustration of professional earnestness.

Now that the Association is out of debt, all fears of the ultimate failure of the institution is at an end. It can't fail now; and the best thing to be done is for the profession in the west and southwest to rally, as one man, to the rescue, and see that the College is properly supported, both with patronage and appliances. The indebtedness being gone, the same spirit, manifested for a year or two longer, will secure a library, apparatus and specimens that any institution might be proud of. Let the good work never stop. Let us remember that this Association has erected the first edifice ever built for the single purpose of dental education. Its members may well be proud of their position. They are each a *founder* of an important institution, that is to take high and permanent rank in the grand race of civilization. Let us support and sustain the old college with a will. It deserves it. In blessing it, we will more than bless ourselves. W.

### MISSISSIPPI VALLEY ASSOCIATION.

This old society held its late annual meeting, in the Ohio Dental College, February 17th; and the attendance, though far short of what it should have been, was quite encouraging. The discussions were far more practical than are usually heard—so *practical* that no written report can do them anything like justice. Those who failed to attend have sustained a loss from which they will never recover. The minutes and report of discussions will show that the Association has the vigor and energy of youth, as well as the ripe sense and maturity of age. We have failed to attend but one meeting of this Association since we became connected with the dental profession, and oh how we do regret that failure! We like all the dental societies; but the old "MISSISSIPPI VALLEY" is our *belle-ideal* of what an association ought to be. W.

### A MEDAL-SOME ASSOCIATION.

THE old Mississippi Valley Association has been, and met, and done it. Each member feeling that he didn't know half as much as he ought to, nor quarter as much as he would like to, with one consent the Association resolved to offer a gold medal for the best Practical Essay on Anæsthetics. The matter was intrusted to a committee of five, who are to examine the essays and make the award, if any worthy of such award is presented. This is well. And now, we hope dentists, physicians, surgeons and chemists will all endeavor to give all the light they can on this great subject. Competition is open to all the world; and we are glad of it. If a John Bull, or a John Chinaman, or even a John Donkey, gives us a better practical essay than any dentist can write, we will hail its production as a blessing to mankind, and an honor to *our country;* for as a professsion, "the world is our country, and mankind our countrymen." We hope to see—to hear, at least, of a number of essays competing for the prize. W.

